# Age-Related Differences in Socio-demographic and Behavioral Determinants of HIV Testing and Counseling in HPTN 043/NIMH Project Accept

**DOI:** 10.1007/s10461-017-1807-5

**Published:** 2017-06-06

**Authors:** N. Salazar-Austin, M. Kulich, A. Chingono, S. Chariyalertsak, K. Srithanaviboonchai, G. Gray, L. Richter, H. van Rooyen, S. Morin, M. Sweat, J. Mbwambo, G. Szekeres, T. Coates, D. Celentano, The NIMH Project Accept (HPTN 043) Study Team, Salim Abdool Karim, Janet Frohlich, Philip Joseph, Thulani Ngubani, Linda Richter, Heidi van Rooyen, Laurie Abler, Suzanne Maman, Audrey Pettifor, Christopher Bamanyisa, Lillianne Chovenye, G. P. Kilonzo, Nora Margaret Hogan, Florence P. Lema, Jessie K. K. Mbwambo, Khalifa M. Mrumbi, Chris Beyrer, David D. Celentano, Becky Genberg, Surinda Kawichai, Benjamin Link, Carla E. Zelaya, Adam W. Carrico, Sebastian Kevany, Gertrude Khumalo-Sakutukwa, Tim Lane, Joanne Mickalian, Simon Morfit, Stephen F. Morin, Wayne Steward, Chonlisa Chariyalertsak, Suwat Chariyalertsak, Surinda Kawichai, Kriengkrai Srithanaviboonchai, Surasing Visrutaratna, Alfred Chingono, Tendayi Jubenkanda, Memory Sendah, Tserayi Machinda, Oliver Murima, Andrew Timbe, Godfrey Woelk, Thomas J. Coates, Agnès Fiamma, Greg Szekeres, Kathryn Curran, Andrew M. Sadowski, Michael Sweat, Basant Singh, Marta I. Mulawa, Deborah Donnell, Susan H. Eshleman, LeTanya Johnson-Lewis, Oliver Laeyendecker, Estelle Piwowar-Manning, Katherine Fritz, Amy Gregowski, Glenda Gray, Sakhile Mhlongo, Precious Modiba, Gavin Robertson, Harry Hausler, Zdenek Hlavka, Daniel Hlubinka, Michal Kulich, Oliver Laeyendecker, Nuala McGrath, James McIntyre

**Affiliations:** 1 Department of Pediatrics, Johns Hopkins School of Medicine, 200 N. Wolfe St Room 3147, Baltimore, MD 21287 USA; 2 Faculty of Mathematics and Physics, Department of Probability and Statistics, Charles University, Prague, Czech Republic; 3 University of Zimbabwe College of Health Sciences, Harare, Zimbabwe; 4 Department of Community Medicine, Chiang Mai University, Chiang Mai, Thailand; 5 South African Medical Research Council, Cape Town, South Africa; 6 University of the Witwatersrand, Johannesburg, South Africa; 7 Human Sciences Research Council, Pretoria, South Africa; 8 Department of Medicine, University of California, San Francisco, USA; 9 Division of Global and Community Health, Medical University of South Carolina, Charleston, SC USA; 10 Muhimbili University of Health and Allied Sciences, Dar es Salaam, Tanzania; 11 UCLA Center for World Health, University of California, Los Angeles, USA; 12 Department of Medicine, University of California, Los Angeles, CA USA; 13 Department of Epidemiology, Johns Hopkins Bloomberg School of Public Health, Baltimore, USA

**Keywords:** Mobile HIV testing and counseling, Youth, High-risk sexual behavior, Project accept, Determinants HTC

## Abstract

Youth represent a large proportion of new HIV infections worldwide, yet their utilization of HIV testing and counseling (HTC) remains low. Using the post-intervention, cross-sectional, population-based household survey done in 2011 as part of HPTN 043/NIMH Project Accept, a cluster-randomized trial of community mobilization and mobile HTC in South Africa (Soweto and KwaZulu Natal), Zimbabwe, Tanzania and Thailand, we evaluated age-related differences among socio-demographic and behavioral determinants of HTC in study participants by study arm, site, and gender. A multivariate logistic regression model was developed using complete individual data from 13,755 participants with recent HIV testing (prior 12 months) as the outcome. Youth (18–24 years) was not predictive of recent HTC, except for high-risk youth with multiple concurrent partners, who were less likely (aOR 0.75; 95% CI 0.61–0.92) to have recently been tested than youth reporting a single partner. Importantly, the intervention was successful in reaching men with site specific success ranging from aOR 1.27 (95% CI 1.05–1.53) in South Africa to aOR 2.30 in Thailand (95% CI 1.85–2.84). Finally, across a diverse range of settings, higher education (aOR 1.67; 95% CI 1.42, 1.96), higher socio-economic status (aOR 1.21; 95% CI 1.08–1.36), and marriage (aOR 1.55; 95% CI 1.37–1.75) were all predictive of recent HTC, which did not significantly vary across study arm, site, gender or age category (18–24 vs. 25–32 years).

**Electronic supplementary material:**

The online version of this article (doi:10.1007/s10461-017-1807-5) contains supplementary material, which is available to authorized users.

## Resumen

Los jóvenes representan una gran proporción de nuevas infecciones por el VIH en todo el mundo, sin embargo, su utilización de las pruebas de detección del VIH y el asesoramiento (HTC) sigue siendo baja. Utilizando la encuesta de población transversal de la población realizada en 2011 como parte del proyecto HPTN 043 / NIMH Project Accept, un ensayo aleatorizado por grupos de movilización comunitaria y HTC móvil en Sudáfrica (Soweto y KwaZulu Natal), Zimbabwe, Tanzania y Tailandia, evaluamos las diferencias relacionadas con la edad entre los determinantes sociodemográficos y conductuales de HTC en los participantes del estudio por brazo de estudio, sitio y sexo. Se desarrolló un modelo multivariado de regresión logística utilizando datos individuales completos de 13,755 participantes con pruebas de VIH recientes (antes de 12 meses) como resultado. Los jóvenes (18-24 años) no eran predictivos de HTC recientes, excepto los jóvenes de alto riesgo con múltiples parejas concurrentes, que eran menos probables (aOR 0,75; IC del 95%: 0,61-0,92) compañero. Es importante destacar que la intervención fue exitosa en hombres con éxito específico en el sitio, desde aOR 1,27 (IC 95% 1,05-1,53) en Sudáfrica hasta aOR 2,30 en Tailandia (IC 95%: 1,85-2,84). Por último, en una amplia gama de contextos, la educación superior (aO 1,67; IC del 95%: 1,42; 1,96); mayor estatus socioeconómico (aOR 1,21; IC del 95%: 1,08-1,36) y matrimonio (aOR 1,55; IC del 95%: 1,37 -1.75) eran todos predictivos de HTC recientes, que no variaron significativamente entre brazo de estudio, sitio, sexo o categoría de edad (18-24 vs 25-32 años).

## Introduction

Youth age 15–24 years account for 42% of new HIV diagnoses worldwide [1]. Uptake of HIV testing and counseling (HTC) by these youth remains inadequate [2, 3]. It is estimated that only 15% of young women and 10% of young men in sub-Saharan Africa know their HIV status [4]. HIV incidence among 15–24 year olds is high, particularly among young women, where 7500 young women are estimated to acquire HIV each week [5]. From 2010 through 2015, UNAIDS estimates only a 6% decline in HIV incidence among females age 15–24 years [5]. Focused HIV prevention on this age group remains a priority given the bold target of reducing the annual rate of new HIV infections among adolescent and young women to under 100,000 by 2020 [5].

HTC is the first step into both the HIV prevention and treatment cascade. HTC not only identifies one’s HIV status, but can also positively influence one’s sexual risk behavior and reduce the likelihood of future HIV acquisition or further transmission [6, 7]. Reported socio-demographic factors associated with HTC include older age, marriage, higher educational status, urban residence, higher socio-economic status (SES), and reporting a single partner [8, 9]. For youth age 15–24 years, studies have shown pregnancy or ever having made someone pregnant, urban residence, higher education (for men), and a higher frequency of clinic visits are all predictors of HTC among South African youth. Additionally, being HIV+ (among men) or knowing someone who has died of AIDS (for men), possessing knowledge of HIV, having had a parental discussion regarding HIV, and participating in HIV prevention programming are also predictors of HIV testing in youth [10, 11]. Youth also report significant psychological barriers to HIV testing including lack of community support and perceived negative attitudes of health care workers [12]. Further understanding the factors that lead youth to undergo HIV testing is critical to creating focused strategies to increase HTC uptake amongst this at-risk population.

National AIDS programs have tried to motivate high-risk youth to regularly undergo HTC, but have not always been successful, especially among adolescents and young adults [5]. Programs have relied on traditional facility-based HTC which has evolved to include both provider-initiated testing and routine, or opt-out, testing. To reach those community members who do not regularly access health care, community-based approaches such as mobile HTC, home-based HTC, and self-testing have been developed [13–19]. For youth, home-based and self-testing strategies may provide increased confidentiality. No matter what method is used, truly supportive services that provide non-judgmental, empathetic counseling services is critical for youth buy-in and their continued access of both HIV prevention and treatment services [20]. While individually these approaches hold promise [16, 21], a combination of approaches will likely be necessary to attain universal HTC coverage to reach 90% of those living with HIV, the goal set by UNAIDS for 2020 [22]. Each of these methods are being used to target youth age 15–24 years, but have not been evaluated to determine their optimal use among this vulnerable and important age group [20]. Further studies are needed to demonstrate acceptability and improvement in HTC uptake for youth [20, 21].

Traditionally, young men have poorly sought out health services. Voluntary medical male circumcision programs have successfully reached over 11 million adolescent boys in Africa since 2008 [5]. UNAIDS aims to use this platform to provide over 90% of men age 10–29 years with customized, age-appropriate health services by 2021 [5].

Project Accept was a community-level cluster randomized trial of a multilevel structural HIV prevention intervention with mobile HTC conducted from 2007 to 2010 across a variety of communities in Thailand, Zimbabwe, Tanzania, and both urban (Soweto) and rural (Vulindlela) South Africa [23, 24]. The primary outcome, community-level HIV incidence, was compared between communities randomized to community-based voluntary counseling and testing (CBVCT) with mobile testing and study-supported stigma-reducing interventions versus traditional facility-based or standard voluntary counseling and testing (SVCT). HIV prevalence varied among study sites from <1% in Thailand to 31% in KwaZulu-Natal, South Africa. An interim evaluation of HTC in 2009, using HTC service utilization data available from Tanzania, Zimbabwe and Thailand, showed 28% of HTC clients in CBVCT communities were receiving repeat HTC and that across three community pairs, HTC uptake was 40% higher among clinic clients in CBVCT communities [25]. This study utilized limited data collected on the subjects who used HTC services and could therefore not fully evaluate socio-demographic and behavioral determinants of HTC. Using the more detailed post-intervention cross-sectional survey data, the primary analysis showed a 25% increase in recent HTC (over the prior 12 months) in CBVCT versus SVCT communities. This increase was more profound for men (45% increase) than women (15% increase) [13]. The diversity of Project Accept sites provides a unique opportunity to evaluate socio-demographic and behavioral determinants of HTC across a broad spectrum of HIV epidemics spanning two continents, four countries, and rural and urban communities, to evaluate which subgroups mobile VCT may be most effective at targeting. This analysis aims to determine age related differences in HTC uptake among HPTN 043 Project Accept post-intervention survey participants, comparing youth age 18–24 years to those 25–32 years, by site, study arm and gender, to inform future strategies to improve HTC uptake among youth.

## Methods

Project Accept was a community-level cluster-randomized trial of community mobilization, mobile HTC, and post-test support services aimed at reducing community-wide HIV incidence and HIV-related stigma conducted during 2007–2010 in 48 communities in Thailand, Zimbabwe, Tanzania, and two sites in South Africa (Vulindlela, KwaZulu Natal and Soweto, Gauteng). Study outcomes were assessed using a cross-sectional, population-based, post-intervention household survey that was conducted from 2009 to 2011. Methodology is described in detail elsewhere [23, 24]. Briefly, men and women ages 18–32 were recruited across all 48 communities, regardless of participation in Project Accept activities or participation in the baseline survey. Using a complete listing of community households, households were randomly selected and visited by interview teams until they attained the pre-specified sample size to assess the primary outcome of community-level HIV incidence [24]. After permission was obtained from the head of the household, eligible household members were then listed, and one was randomly selected for participation in a detailed socio-demographic and behavioral assessment using the Kish grid method and consented for participation. All surveys and participant consents were approved by all involved US institutions and local ethics committees. This sub-study was considered IRB-exempt by the Johns Hopkins School of Public Health Institutional Review Board.

### Measures

Survey questions were designed collaboratively with all sites. HIV testing was evaluated first by having ever been tested, second by the timing of that testing (>3 years, 1–3 years or <1 year) and finally by the frequency of testing (once versus repeated). Recent HIV testing, defined as HTC over the prior 12 months, was used as the outcome to assess for both the intervention’s effect and the need for recurrent HTC among high-risk sub-groups.

Socio-demographic factors included age, gender, education, marital status, SES, and employment. Marital status was classified as currently married or unmarried. SES was assessed using site-specific, local definitions compositing income and household assets and classified as low, medium or high. Behavioral factors included sexual activity, number of partners, frequency of sexual activity, and frequency of condom use. Recent sexual risk behavior was assessed during the 6 months prior to survey participation.

### Statistical Analyses

Out of the original 14,291 post-intervention survey participants who completed the detailed socio-demographic and behavioral interview, 536 subjects with incomplete data were excluded. All analyses were performed on the remaining 13,755 participants with complete data. Multivariate logistic regression models were used with recent testing (defined as HIV testing in the prior 12 months) as the outcome. Significance of predictors was assessed by likelihood ratio tests at the 0.05 level. First, a base model including site, gender, intervention, age category (18–24 vs. 25–32 years), and their significant interactions was built. Next, individual socio-demographic and behavioral factors were added one by one to the base model, including its interactions with gender, age, intervention, and site. The final model was obtained by simultaneously adding all significant factors and interactions from these smaller models to the base model and removing all insignificant terms. Two versions of the final model were fitted: unadjusted for community effects (59 parameters) and adjusted for community effects (97 parameters). Adjustment for community effects was done by contrasts that summed to zero within each site-by-intervention combination so that overall site and intervention effects would not be affected by adjustment for community. Confidence intervals are based on Wald tests. The analysis was performed in the R software environment.

## Results

### Study Population

There were 13,755 participants age 18–32 years who completed Project Accept’s detailed socio-demographic and behavioral post-intervention survey and had complete data in all variables considered for analysis (Table 1). Among all survey participants, 34.6% reported at least one recent HIV test in CBVCT communities and 29.3% in SVCT communities (Table 2). Among youth participants age 18–24 years, 31.8% reported recent HIV testing in CBVCT communities and 26.9% in SVCT communities (Table 3). Only 22.0 and 16.1% of young men (18–24 years) reported recent testing in CBVCT and SVCT communities, respectively. Testing rates in young women (18–24 years) were at least twice as large (Table 3).

**Table 1:** Study population: Participant characteristics of HPTN 043 project accept post-intervention cross-sectional community survey

Site	Thailand		Zimbabwe		Tanzania		KwaZulu-Natal		Soweto		All sites	
Intervention	CBVCT	SVCT	CBVCT	SVCT	CBVCT	SVCT	CBVCT	SVCT	CBVCT	SVCT	CBVCT	SVCT
n	1505 (%)	1569 (%)	1248 (%)	1243 (%)	1386 (%)	1379 (%)	1293 (%)	1234 (%)	1439 (%)	1459 (%)	6871 (%)	6884 (%)
Gender												
Male	49.1	51.1	43.7	46.2	41.9	43.1	42.5	41.2	45.2	44.7	44.6	45.5
Female	50.9	48.9	56.3	53.8	58.1	56.9	57.5	58.8	54.8	55.3	55.4	54.5
Age group												
18–24	45.6	48.1	48.1	49.1	41.9	39.0	58.5	63.4	52.5	54.6	49.2	50.6
25–32	54.4	51.9	51.9	50.9	58.1	61.0	41.5	36.6	47.5	45.4	50.8	49.4
Education (years)												
0–5	33.5	19.5	3.5	3.5	23.4	24.6	2.6	2.6	0.5	1.0	13.3	10.7
6–9	37.7	41.4	40.1	39.3	62.1	65.8	9.1	17.3	6.5	5.3	31.2	33.9
10–12	20.7	26.3	51.4	50.1	13.6	8.6	85.0	76.2	70.0	68.1	47.3	44.9
13 or more	8.1	12.8	4.9	7.1	0.9	1.0	3.3	4.0	23.0	25.7	8.3	10.6
SES group												
Low	37.9	27.9	23.8	28.6	31.2	35.8	17.5	22.1	5.8	4.7	23.4	23.6
Medium	30.2	31.9	29.3	25.0	46.6	45.5	65.0	61.9	62.1	65.9	46.6	46.0
High	32.0	40.2	46.9	46.4	22.2	18.8	17.5	16.0	32.1	29.3	30.0	30.4
Employment												
Yes	88.7	85.7	57.0	63.4	63.0	64.8	39.6	38.8	58.2	58.4	62.1	63.3
No	11.3	14.3	43.0	36.6	37.0	35.2	60.4	61.2	41.8	41.6	37.9	36.7
Marital status												
Married	55.7	49.2	53.1	54.7	51.2	53.4	2.6	2.0	8.8	7.3	34.5	33.7
Unmarried	44.3	50.8	46.9	45.3	48.8	46.6	97.4	98.0	91.2	92.7	65.5	66.3
Ever had sex												
Yes	87.4	84.0	85.7	87.5	90.5	92.8	87.6	85.2	91.4	91.6	88.6	88.2
No	12.6	16.0	14.3	12.5	9.5	7.2	12.4	14.8	8.6	8.4	11.4	11.8

*n* number of subjects participating in the post-intervention survey, *CBVCT* community-based voluntary counseling and testing, *SVCT* standard voluntary counseling and testing

**Table 2:** HIV testing history: reported HIV testing history in HPTN 043 project accept post-intervention survey

Site	Thailand		Zimbabwe		Tanzania		KwaZulu-Natal		Soweto		All sites	
Intervention	CBVCT	SVCT	CBVCT	SVCT	CBVCT	SVCT	CBVCT	SVCT	CBVCT	SVCT	CBVCT	SVCT
n	1505 (%)	1569 (%)	1248 (%)	1243 (%)	1386 (%)	1379 (%)	1293 (%)	1234 (%)	1439 (%)	1459 (%)	6871 (%)	6884 (%)
No test	36.5	55.1	50.8	57.2	33.1	38.4	40.4	46.8	38.2	39.3	39.5	47.3
Test more than 3 years ago	19.2	16.8	9.1	9.7	20.6	19.6	12.8	13.0	13.1	14.3	15.2	14.8
Test 1–3 years ago	20.1	13.6	7.8	6.8	10.0	8.3	6.3	4.6	8.1	8.2	10.7	8.6
Once in past year	20.9	12.0	19.9	19.1	19.5	21.5	20.3	18.6	20.7	21.9	20.3	18.5
Repeated in past year	3.3	2.5	12.5	7.3	16.8	12.3	20.0	16.9	19.9	16.3	14.3	10.8

*n* number of participants with known testing status, *CBVCT* community-based voluntary counseling and testing, *SVCT* standard voluntary counseling and testing

**Table 3:** Percent testing in the last 12 months by age and gender

Site	Thailand		Zimbabwe		Tanzania		KwaZulu-Natal		Soweto		All sites	
Intervention	CBVCT	SVCT	CBVCT	SVCT	CBVCT	SVCT	CBVCT	SVCT	CBVCT	SVCT	CBVCT	SVCT
n	1505 (%)	1569 (%)	1248 (%)	1243 (%)	1386 (%)	1379 (%)	1293 (%)	1234 (%)	1439 (%)	1459 (%)	6871 (%)	6884 (%)
Gender												
Male	19.6	10.1	24.6	14.8	25.3	19.0	31.8	22.0	24.0	25.6	24.7	17.8
Female	28.6	19.0	38.4	36.3	44.2	45.0	46.7	45.0	54.2	48.5	42.6	38.9
Age group												
18–24	23.3	12.3	28.0	23.3	30.5	30.1	38.3	33.9	37.0	34.3	31.8	26.9
25–32	24.9	16.4	36.4	29.4	40.5	36.1	43.3	38.5	44.5	43.1	37.3	31.8
Gender and age												
Men 18–24 years	19.0	9.2	19.4	12.1	22.9	19.3	30.0	20.2	18.8	21.1	22.0	16.1
Men 25–32 years	20.2	11.1	31.6	18.2	27.5	18.8	34.8	26.1	30.3	31.8	27.8	19.9
Women 18–24 years	28.0	16.1	37.4	35.6	37.3	41.3	45.1	45.0	53.2	46.0	41.1	37.5
Women 25–32 years	29.1	21.4	39.1	36.8	48.5	46.8	48.8	45.1	55.3	51.2	43.8	40.1

*n* number of participants with known age, gender, and testing status, *CBVCT* community-based voluntary counseling and testing, *SVCT* standard voluntary counseling and testing

### Socio-Demographic and Behavioral Characteristics of Recent Testing

A higher proportion of youth (18–24 years) in CBVCT communities underwent recent HIV testing independent of site, gender, and all measured socio-demographic and behavioral covariates, (Table 4), including low-risk groups such as those reporting never having had sex. Higher percentages of recent HCT were observed among participants who were sexually active, had at least 10 years of education, or lived in households with medium or high SES.

**Table 4:** Percent testing in the last 12 months by explanatory factors

Gender, age	Men 18–24 years		Men 25–32 years		Women 18–24 years		Women 25–32 years	
Intervention	CBVCT	SVCT	CBVCT	SVCT	CBVCT	SVCT	CBVCT	SVCT
n	1643 (%)	1734 (%)	1422 (%)	1396 (%)	1738 (%)	1747 (%)	2068 (%)	2007 (%)
Years of education								
0–5	17.7	11.8	20.2	13.0	29.2	28.9	34.9	29.1
6–9	18.2	11.1	27.7	15.5	35.7	35.0	41.7	39.3
10–12	24.3	18.4	30.6	26.5	44.8	39.7	49.7	44.9
13 or more	24.0	23.2	30.4	22.8	46.6	38.2	46.2	43.4
SES group								
Low	18.1	11.5	23.0	14.4	39.2	33.1	38.4	36.5
Medium	23.2	17.8	28.0	22.0	42.0	41.1	45.4	39.3
High	22.6	16.8	31.6	20.9	40.9	34.5	46.3	44.8
Employment (has income from work)								
Yes	23.5	16.2	27.8	19.1	41.5	36.8	43.6	40.1
No	20.0	16.0	28.0	24.6	40.7	38.1	44.3	40.2
Marital status								
Married	33.5	26.2	27.8	17.6	40.1	38.3	41.5	38.4
Unmarried	20.7	15.0	27.9	21.5	41.5	37.2	46.6	42.2
Ever had sex								
Yes	24.2	18.1	28.4	20.2	46.5	42.7	44.2	40.6
No	14.5	9.5	14.8	13.8	17.1	13.7	22.9	21.3
Number of partners in the last 6 months								
0	16.9	11.4	23.0	12.3	32.5	30.8	48.3	44.0
1	29.5	22.3	28.1	22.0	46.3	41.3	42.4	39.0
2	19.7	17.9	30.4	22.9	46.9	41.2	53.3	48.7
3 or more	23.5	17.9	36.3	25.9	60.0	63.6	55.6	33.3

*n* number of participants of given gender and age range who reported testing status, *CBVCT* community-based voluntary counseling and testing, *SVCT* standard voluntary counseling and testing

### Socio-demographic Predictors of Recent Testing

The intervention’s effect on recent HIV testing was highly significant and varied by both site and gender, but not age (Table 5). The strongest effect was seen in Thailand for men (aOR 2.30; 95% CI 1.85–2.84) and, to a lesser effect, in non-pregnant women (aOR 1.92; 95% CI 1.56–2.36). The intervention had a minimal effect on HIV testing among non-pregnant women in Tanzania (aOR 1.09; 95% CI 0.91–1.30), KwaZulu Natal, South Africa (aOR 1.12; 95% CI 0.91–1.34) and Soweto, South Africa (aOR 1.06; 95% CI 0.89–1.26). Overall, intervention effects were even weaker among women reporting current pregnancy than among non-pregnant women (aOR 0.56, 95% CI 0.39–0.80).

**Table 5:** Intervention effect on recent HIV testing (past 12 months) by gender and site: results of multivariate logistic regression model

Site	Thailand	Zimbabwe	Tanzania	South Africa KwaZulu-Natal	South Africa Soweto
n = 13,755	n = 3074	n = 2491	n = 2765	n = 2527	n = 2898
Gender	aOR [95% CI] p	aOR [95% CI] p	aOR [95% CI] p	aOR [95% CI] p	aOR [95% CI] p
Males	2.30 [1.85–2.84] p<0.001	1.60 [1.30–1.97] p<0.001	1.31 [1.07–1.59] p = 0.007	1.34 [1.10–1.64] p = 0.004	1.27 [1.05–1.53] p = 0.012
Females (non-pregnant)	1.92 [1.56–2.36] p < 0.001	1.33 [1.10–1.62] p = 0.004	1.09 [0.91–1.30] p = 0.337	1.12 [0.93–1.34] p = 0.218	1.06 [0.89–1.26] p = 0.506

Adjusted odds ratio (aOR) for recent HIV testing in CBVCT communities relative to SVCT communities by site and gender with 95% confidence intervals [95% CI], and p-values (p) for no intervention effect

Overall intervention effects on recent HIV testing were previously reported [13]

aORs were adjusted for marital status, education, employment, SES, sexual activity and condom use

Age was not directly associated with recent testing when socio-demographic and behavioral factors were taken into account. Participants who were not married, including those who reported being single, divorced, separated or widowed, were less likely (aOR 0.64, 95% CI 0.57–0.73) to have been recently tested than participants who reported being currently married. More educated participants, particularly those who had attended at least 10 years of schooling (aOR 1.67, 95% CI 1.42–1.96 relative to a maximum of 5 years of schooling), those who earned money from employment (aOR 1.16, 95% CI 1.06–1.27), and those with the highest site-specific SES (aOR 1.21, 95% CI 1.08–1.36 compared to lowest SES) were all more likely to have tested in the prior 12 months (Table 6).

**Table 6:** Socio-demographic predictors of recent HIV testing (past 12 months): results of multivariate logistic regression model

Socio-demographic factor	All participants (n = 13,755)		
	aOR	95% CI	p
Years of education			**<0.0001**
0–5 (baseline level)	1.00	–	–
6–9	1.28	1.11–1.48	<0.001
10–12	1.67	1.42–1.96	<0.001
13 or more	1.68	1.38–2.04	<0.001
SES group			**0.0008**
Low (baseline level)	1.00	–	–
Medium	1.03	0.93–1.15	0.52
High	1.21	1.08–1.36	0.001
Employment (has income from work)			**0.001**
Yes (baseline level)	1.00	–	–
No	0.86	0.79–0.94	0.001
Marital status			**<0.001**
Unmarried (baseline level)	1.00	–	–
Married	1.55	1.37–1.75	<0.001

Adjusted odds ratio (aOR) for recent HIV testing relative to the baseline level with 95% confidence intervals [95% CI], and p-values (p). Bold p-values test the overall effect or the factor. aORs were adjusted for site, intervention, gender, age, and other socio-demographic and behavioral factors

Importantly, the effects of socio-demographic factors on recent HIV testing did not vary significantly between the five sites, between intervention and control communities, between genders, or between age categories (18–24 vs. 25–32 years).

### Behavioral Predictors of Recent HIV Testing

Reporting multiple partners in the past 6 months was the only factor that had an effect on testing rates that varied according to age. Youth, age 18–24 years, with multiple partners were less likely to test (aOR 0.75, 95% CI 0.61–0.92) than youth reporting a single partner. However, older participants, age 25–32 years, with multiple partners were not less likely to test (aOR 1.12, 95% CI 0.91–1.36) compared to older participants with a single partner (Table 7). Men who were not sexually active in the 6 months prior to the interview were less likely to report recent testing (aOR 0.56, 95% CI 0.45–0.69). In sexually active women, testing rates were not associated with patterns of sexual activity (aOR 1.03, 95% CI 0.86–1.25) (Table 7).

**Table 7:** Behavioral predictors of recent HIV testing (past 12 months): results of multivariate logistic regression model

Behavioral factor	Age 18–24 (n = 6862)			Age 25–32 (n = 6893)		
	aOR	95% CI	p	aOR	95% CI	p
Number of partners in the last six months			**0.007**			**0.29**
1 partner (baseline level)	1.00	–	–	1.00	–	–
multiple partners	0.75	0.61–0.92	0.007	1.12	0.91–1.36	0.29

	Men (n = 6195)			Women (n = 7560)		
Ever had sex			**<0.001**			**<0.001**
Last active <6 months ago (baseline)	1.00	–	–	1.00	–	–
Last active > 6 months ago	0.56	0.45–0.69	<0.001	1.03	0.86–1.25	0.73
Never been active	0.42	0.32–0.54	<0.001	0.26	0.20–0.33	<0.001
Condom use over prior 30 days			**0.003**			**<0.001**
Always (baseline level)	1.00	–	–	1.00	–	–
Almost always	1.01	0.76–1.34	0.95	1.26	0.95–1.68	0.11
Sometimes	0.62	0.47–0.82	0.001	1.07	0.84–1.36	0.60
Rarely	0.85	0.65–1.11	0.24	0.81	0.64–1.04	0.09
Never	0.81	0.66–0.99	0.04	0.71	0.59–0.84	<0.001

Effect of sexual activity and condom use varied with gender. Effect of number of partners varied with age

Adjusted odds ratio (aOR) for recent HIV testing relative to the baseline level with 95% confidence intervals [95% CI], and p-values (p). Bold p-values test the overall effect or the factor. aORs were adjusted for site, intervention, gender, age, and other socio-demographic and behavioral factors

In sexually active participants, higher rates of condom use were generally associated with higher rates of recent testing, but the effect was somewhat different in men than in women. Male participants who use condoms about half of the time were less likely (aOR 0.62; 96% CI 0.47–0.82) to have been recently HIV tested compared to regular male condom users. Female participants who used condoms about half the time were not less likely (aOR 1.07; 95% CI 0.84–1.36) to have recently been HIV tested compared to regular female condom users. Both male (aOR 0.81; 95% CI 0.66–0.99) and female (aOR 0.71; 95% CI 0.59–0.84) participants who never used condoms were less likely to have tested for HIV compared to regular condom users.

The reported results were adjusted for site, but not community-level effects. When fixed community effects were added to the model, they were highly significant, but the results for all other predictors were very similar (supplemental table).

## Discussion

This sub-analysis of Project Accept data demonstrates community mobilization, stigma reduction and mobile HTC are successful at reaching many at-risk demographics including youth age 18–24 years and men. Nearly one in three youth age 18–24 years in CBVCT communities reported recent HIV testing. While the proportion of youth tested falls short of UNAIDS’ 90–90–90 goal of having 90% of people living with HIV diagnosed, given the study occurred in the early ARV treatment era, having one-third of youth HIV tested is an important start to making HTC normative and serves as an important baseline for future comparisons in this vulnerable age group.

Our analysis overestimates the at-risk population given our inability to account for those participants who do not require ongoing repeat testing due to a long-standing HIV+ status. We therefore underestimate the proportion of at-risk participants who have undergone recent HIV testing. This would disproportionately affect both the older age group, as HIV prevalence increases with age, and exceptionally high-prevalence communities, including both South African sites [13].

Across all sites, CBVCT appears to be successful in getting young men to test, a group that does not traditionally utilize fixed clinics. Women access fixed clinics for antenatal care, likely explaining the higher proportion of recent HTC among women and the smaller relative impact of CBVCT. Teasing out the effect of CBVCT on women was not possible because previous pregnancy was not assessed in the post-intervention survey and we were therefore unable to isolate the effect of pregnancy on prior HIV testing. Community-based mobile testing may be a particularly useful strategy in the identification of HIV among some high-risk young men, who may be critical to HIV transmission dynamics.

In our model, youth (age 18–24 years) was found to modify the effect of having multiple partners on recent HIV testing across all sites, in both intervention and control communities, and among both genders. Youth with multiple partners in the last six months were less likely to have recently tested for HIV. This may be explained by youth’s heightened risk taking behaviors that are partially controlled by the prefrontal cortex [26] which only reaches full maturity around 24 years of age [27]. However, prior adult and adolescent studies in developed countries have shown those individuals with multiple partners were more likely to have been HIV tested [28–30]. The studies focused exclusively on youth suggested the health care provider plays a critical role in providing influential information and counseling to at-risk youth [29, 30]. Health care workers in the developing world may be undertrained in providing youth-friendly services including a supportive, non-judgmental and reassuringly confidential approach to counseling youth [4, 12, 20, 31, 32]. Addressing this gap in youth-services may be critical in improving HTC among high-risk youth.

HPTN 043 Project Accept includes data from 48 paired communities, across 5 sites, 4 countries and 2 continents, reflecting the diversity of the HIV epidemic and the driving forces behind their local epidemics. This helps to explain the variable intervention effect across different sites and subpopulations. Even still, across all sites, community mobilization and mobile testing increased youth HIV testing rates across all assessed socio-demographic and behavioral subgroups. Similar to studies in the pre-treatment era [9], higher education, higher SES, income and marriage all remain predictors of HTC uptake across all five sites, despite the diversity among their cultures and HIV epidemics. While CBVCT was successful at increasing testing, other HTC interventions may be necessary to attain universal testing and reach high-risk groups with persistently poor rates of HTC including those populations that are unmarried, less educated, and of lower socio-economic status [33, 34]. For youth, addressing their age-specific concerns is important. The provision of respectful and supportive counseling in an accepting atmosphere is critical. Confidentiality can be addressed through self-testing. A comfortable testing environment outside of the facility including at social centers, or providing home-based and mobile HTC at youth-targeted community events are important [20].

Given multiple layers of clustering within site, community pairs, villages and households, we were unable to model correlations between individuals using logistic regression with random effects. The most important level of clustering (communities) was added to the model as fixed effects. This limits generalizability to other communities or even other individuals in these communities. Though estimated parameters should remain similar, the calculated 95% confidence intervals may be too narrow. However, the strongest highly significant effects in our model should not be affected by this limitation.

In our model, lack of HIV testing among youth 18–24 years was attributable to other socio-demographic and behavioral characteristics known to be associated with lower HIV testing rates in all age groups. Importantly, community-based mobile testing did improve HTC uptake in youth including men, who traditionally demonstrate poor utilization of facility-based HTC. Youth with higher sexual risk were less likely to have obtained recent HIV testing. Incorporating this knowledge into youth-friendly HIV prevention messaging, counseling and services is important.

## Conclusions

Mobile HTC was successful in reaching youth, age 18–24 years, an important at-risk population. This was particularly true for young men. Youth 18–24 years with high-risk sexual behavior, including multiple concurrent partners, accessed HTC less commonly in all communities including those with mobile and facility-based HTC. As HTC remains a necessary gateway to both treatment and prevention services; improving the quality of youth-based HTC services may not only improve access for the majority of youth, but also youth with high-risk behavior.

## Electronic supplementary material

Below is the link to the electronic supplementary material.

Supplementary material 1 (DOCX 25 kb)
