# Supplementary material for: Age-Related Differences in Socio-demographic and Behavioral Determinants of HIV Testing and Counseling in HPTN 043/NIMH Project Accept
Source: AIDS Behav. 2017 Jun 6;22(2):569–79. doi: 10.1007/s10461-017-1807-5 (PMC5718984; doi:10.1007/s10461-017-1807-5)
Supplement: Supplementary file 1 — Supplementary material 1 (DOCX 25 kb) [file 10461_2017_1807_MOESM1_ESM.docx]

**Table S5:** Intervention effect on recent HIV testing (past 12 months) by gender and site: results of multivariate logistic regression model adjusted for community effects.

| **Site** | **Thailand** | | | **Zimbabwe** | | | **Tanzania** | **South Africa KwaZulu-Natal** | | | **South Africa Soweto** | |
| --- | --- | --- | --- | --- | --- | --- | --- | --- | --- | --- | --- | --- |
| n=13,755 | n=3074 | | | n=2491 | | | n=2765 | n=2527 | | | n=2898 | |
| **Gender** | *aOR  [95% CI] p* | | | *aOR  [95% CI] p* | | | *aOR  [95% CI] p* | *aOR  [95% CI] p* | | | *aOR  [95% CI] p* | |
| *Males* | 2.27  [1.82 - 2.83] p<0.001 | 1.57  [1.27 - 1.94] p<0.001 | | | | 1.28 [1.05 - 1.56] 0.0139  0.014 | | | 1.33 [1.09 - 1.63] 0.006 | 1.30 [1.07 - 1.57] 0.008 | | |
| *Females   (non-pregnant)* | 1.92 [1.55 - 2.38] p<0.001 | 1.33 [1.09 - 1.62] 0.005 | | | | 1.08 [0.91 - 1.30] 0.378 | | | 1.13 [0.93 - 1.36] 0.211 | 1.10 [0.92 - 1.31] 0.304 | | |
|  | | |  | |  | | |  | | | |  |

Adjusted odds ratio (aOR) for recent HIV testing in CBVCT communities relative to SVCT communities by site and gender with 95% confidence intervals [95% CI], and p-values (p) for no intervention effect.

Overall intervention effects on recent HIV testing were previously reported (13).

aORs were adjusted for community, marital status, education, employment, SES, sexual activity and condom use.

**Table S6a:** Socio-demographic predictors of recent HIV testing (past 12 months): results of multivariate logistic regression model adjusted for community effects.

|  | **All Participants (n=13,755)** | | |
| --- | --- | --- | --- |
| **Socio-demographic factor** | *aOR* | *95% CI* | *p* |
| Years of education |  |  | **< 0.0001** |
| *0-5 (baseline level)* | 1.00 | — | — |
| *6-9* | 1.32 | 1.14-1.53 | <0.001 |
| *10-12* | 1.72 | 1.46-2.02 | <0.001 |
| *13 or more* | 1.79 | 1.46-2.19 | <0.001 |
| SES Group |  |  | **0.0035** |
| *Low (baseline level)* | 1.00 | — | — |
| *Medium* | 1.06 | 0.96-1.18 | 0.261 |
| *High* | 1.21 | 1.08-1.36 | 0.002 |
| Employment (has income from work) |  |  | **0.001** |
| *Yes (baseline level)* | 1.00 | — | — |
| *No* | 0.86 | 0.79-0.94 | 0.001 |
| Marital Status |  |  | **< 0.001** |
| *Unmarried (baseline level)* | 1.00 | — | — |
| *Married* | 1.58 | 1.39-1.79 | <0.001 |

Adjusted odds ratio (aOR) for recent HIV testing relative to the baseline level with 95% confidence intervals [95% CI], and p-values (p). Bold p-values test the overall effect or the factor. aORs were adjusted for community, site, intervention, gender, age, and other sociodemographic and behavioral factors.

**Table S6b:** Behavioral predictors of recent HIV testing (past 12 months): results of multivariate logistic regression model adjusted for community effects. Effect of sexual activity and condom use varied with gender. Effect of number of partners varied with age.

|  | **Age 18-24 (n=6,862)** | | | | **Age 25-32 (n=6,893)** | | | | |
| --- | --- | --- | --- | --- | --- | --- | --- | --- | --- |
| **Behavioral factor** | *aOR* | *95% CI* | | *p* | *aOR* | *95% CI* | | | *p* |
| Number of partners in the last six months |  |  | | **0.004** |  |  | | | **0.30** |
| *1 partner (baseline level)* | 1.00 | — | | — | 1.00 | — | | | — |
| *multiple partners* | 0.73 | 0.59-0.90 | | 0.004 | 1.11 | 0.91-1.36 | | | 0.30 |
|  | **Men (n=6,195)** | | | | **Women (n=7,560)** | | | | |
| **Behavioral factor** | *OR* | *95% CI* | *p* | | *OR* | | *95% CI* | *p* | |
| Ever Had Sex |  |  | **< 0.001** | |  | |  | **< 0.001** | |
| *Last active < 6 months ago (baseline)* | 1.00 | — | — | | 1.00 | | — | — | |
| *Last active > 6 months ago* | 0.55 | 0.44-0.68 | < 0.001 | | 1.20 | | 0.84-1.23 | 0.87 | |
| *Never been active* | 0.40 | 0.31-0.52 | < 0.001 | | 0.25 | | 0.19-0.32 | < 0.001 | |
| Condom Use over prior 30 days |  |  | **0.002** | |  | |  | **< 0.001** | |
| *Always (baseline level)* | 1.00 | — | — | | 1.00 | | — | — | |
| *Almost always* | 0.99 | 0.75-1.32 | 0.97 | | 1.21 | | 0.90-1.61 | 0.20 | |
| *Sometimes* | 0.61 | 0.46-0.81 | 0.001 | | 1.06 | | 0.83-1.35 | 0.65 | |
| *Rarely* | 0.87 | 0.66-1.15 | 0.33 | | 0.82 | | 0.65-1.05 | 0.12 | |
| *Never* | 0.79 | 0.64-0.97 | 0.026 | | 0.71 | | 0.59-0.84 | < 0.001 | |

Adjusted odds ratio (aOR) for recent HIV testing relative to the baseline level with 95% confidence intervals [95% CI], and p-values (p). Bold p-values test the overall effect or the factor. aORs were adjusted for community, site, intervention, gender, age, and other sociodemographic and behavioral factors.
